# Associations between childhood maltreatment and DNA methylation of the oxytocin receptor gene in immune cells of mother–newborn dyads

**DOI:** 10.1038/s41398-021-01546-w

**Published:** 2021-09-01

**Authors:** Laura Ramo-Fernández, Anja M. Gumpp, Christina Boeck, Sabrina Krause, Alexandra M. Bach, Christiane Waller, Iris-Tatjana Kolassa, Alexander Karabatsiakis

**Affiliations:** 1 Clinical & Biological Psychology, Institute of Psychology and Education, Ulm University, Ulm, Germany; 2 Psychosomatic Medicine and Psychotherapy, University Hospital Ulm, Ulm, Germany; 3 Department of Psychosomatics and Psychotherapeutic Medicine, Paracelsus Medical Private University of Nueremberg, Nueremberg, Germany; 4 Department of Clinical Psychology II, Institute of Psychology, University of Innsbruck, Innsbruck, Austria

**Keywords:** Clinical genetics, Physiology, Human behaviour

## Abstract

The neuropeptide oxytocin (OXT) and its receptor (OXTR) modulate interpersonal relationships, particularly mother–child interactions. DNA methylation (DNAm) changes of the *OXTR* gene were observed in individuals who experienced Childhood Maltreatment (CM). A modulatory role of single nucleotide polymorphisms (SNP) within *OXTR* in association with CM on the regulation of OXTR was also postulated. Whether these CM-induced epigenetic alterations are biologically inherited by the offspring remains unknown. We thus investigated possible intergenerational effects of maternal CM exposure on DNAm and *OXTR* gene expression, additionally accounting for the possible influence of three SNP: rs53576 and rs2254298 (*OXTR* gene), and rs2740210 (*OXT* gene). We used the *Childhood Trauma Questionnaire* to classify mothers into individuals with (CM+) or without CM (CM−). Maternal peripheral immune cells were isolated from venous blood (*N* = 117) and fetal immune cells from the umbilical cord (*N* = 113) after parturition. DNA methylation was assessed using MassARRAY. Taqman assays were performed for genotyping and gene expression analyses. Among mothers, CM was not associated with *OXTR* mean methylation or gene expression. However, four CpG sites showed different methylation levels in CM− compared to CM+. In mothers, the *OXTR* rs53576 and *OXT* rs2740210 allelic variations interacted with CM load on the *OXTR* mean methylation. Maternal and newborns’ mean methylation of *OXTR* were positively associated within CM− dyads, but not in CM+ dyads. We show gene×environment interactions on the epigenetic regulation of the oxytocinergic signaling and show the intergenerational comparability of the *OXTR* DNAm might be altered in infants of CM+ mothers.

## Introduction

Childhood maltreatment (CM) includes experiences of physical, sexual and/or emotional abuse, as well as physical and emotional neglect during childhood and adolescence [1]. CM often occurs repetitively or even chronically and therefore, constitutes a major threat to the physical, mental, and emotional development of a child [2–5]. Consequences of CM can last into adulthood [2–6], presumably via epigenetic mechanisms, e.g. via CM-associated alterations in DNA methylation (DNAm). Higher DNAm can result in less gene transcription activity, especially when DNAm occurs in CpG-rich regions within the gene promoter [7]. Such gene-expression changes were suggested to be one central mechanism by which the individual’s physical and emotional systems adapt to stressors like CM [8]. CM-associated changes in the epigenetic regulation of gene products related to physiological stress responses were already reported [9–16]. In contrast, the effects of CM on emotion dysregulation seem to be buffered by the oxytocinergic system [17] via its key players, the neuropeptide oxytocin (OXT) and its receptor (OXTR).

The oxytocinergic system is considered a buffering factor of the long-term consequences of CM on psychological and physical health for several reasons: First, OXT activity is involved in fostering interpersonal relationships (e.g. reproductive behavior, mother–child bonding; [18]). Second, patients with higher OXT plasma levels show fewer symptoms of major depression, post-partum depression, and anxiety symptoms [19, 20]. Third, alterations in oxytocin were also reported in the central nervous system. For example, women with a history of child abuse showed less OXT concentrations in cerebrospinal fluid [21]. On a biomolecular level, we previously reported that OXT levels modulate the physiological impact of CM on immunocellular bioenergetics and telomere length stability [22, 23]. Moreover, the oxytocinergic system might buffer the negative impact of adverse early life experiences and CM on the maladaptive changes in HPA-axis functioning [23]. Finally, OXT has immune-modulating effects (reviewed in [24]). There is cumulating evidence for the impact of CM on the immunoregulatory functions of the oxytocinergic system. For example, the OXTR density in peripheral blood mononuclear cells (PBMC) was reduced with increasing severity of CM experiences [25]. In the context of DNAm, higher methylation of the *OXTR* gene was associated with depression [19, 26, 27] and early life adversity [28, 29], suggesting that DNAm of the *OXTR* might contribute to the pathophysiological transition from chronic stress (i.e., CM) to stress-related pathology. Taken together, these results demonstrate a complex relationship between the oxytocinergic system and early life stress, which might affect *OXTR* regulation not only on an epigenetic level but also on a physiological and behavioral level.

In the last years, traumatic stress research work has focused on the investigation of biological and physiological associations and the potential mechanisms for direct, intergenerational transmission of CM-associated biological changes. If such transmission occurs, inherited changes can impact the development, health, behavior, as well as the psychopathological risk of the non-affected offspring. Based on intergenerational observations, the long-neglected evolutionary theory of Lamarckian inheritance is currently being re-discussed. Epigenetic modifications, and the consequent regulation of the gene expression, offer a plausible mechanism for acquired biological traits and behavioral adaptations to endure throughout generations [30]. Here, the oxytocinergic system has been proposed as a possible mediator of these CM-related intergenerational transmission effects, mainly because it is a key modulator of parenting behavior, attachment, and risk of post-partum depression [31–33]. The associations of CM-linked methylation changes in the *OXTR* gene with translational consequences on maternal behavior, maternal agression, and mother–child bonding may contribute to an intergenerational transmission or direct inheritance of CM-related effects (for a review see ref. [34]). Indeed, children of mothers with CM have a higher risk of emotional and behavioral problems [35]; and newborns of mothers who had been socially isolated during the second pregnancy trimester exhibited less methylated *OXTR* in cord blood cells [36].

Stress research also started to address the relevance of genetic variance for the intergenerational transmission of CM consequences; especially focusing on gene×environment (G×E) interactions, which occur when two or more genotypes respond to environmental challenges with different sensitivity, specificity, or physiological kinetics [17, 34, 37]. Depending on the individual genotype of genes contributing to the oxytocin-signaling pathway, which affects the release of OXT into the bloodstream and the binding efficacy of OXT to its receptor (OXTR), CM experiences might affect maternal oxytocin-related signaling differently. This phenomenon is further discussed in the “differential susceptibility” hypothesis, which suggests that genotypes that predispose to pathology in the general population when associated with adverse events can exert protective effects when associated with favorable environmental conditions, particularly in relation to positive parenting. This is different from the concept of resilience, where genotypes are largely unresponsive to environmental contingencies [38, 39]. This theory explains why genotypes associated with susceptibility have remained throughout evolution. In the context of epigenetic modifications, some single nucleotide polymorphisms (SNPs) in oxytocinergic genes may, in combination with CM, be associated with molecular changes of the oxytocinergic system regulation that might confer adaptation in such challenging early life environment (reviewed in [40]). Similarly, the transmission of biological associations of maternal CM experiences may occur depending on the genotype susceptibility of their offspring. On the behavioral scale, biological alterations might lead to changes in parenting and bonding towards their newborns [41]. Therefore, it is of importance to additionally account for allelic variations in the context of intergenerational studies [38].

Research on oxytocin-signaling pathway genes has mostly focused on the sites rs53576 (G/A) and rs2254298 (G/A), both located within intron 3 of the *OXTR* gene, and on rs2740210 (A/C), located within the *OXT* gene. These three SNPs are involved in several G×E interactions that modulate the risk for psychopathology (reviewed in [42]). The GG genotype of *OXTR* rs53576 in combination with CM was linked to emotion dysregulation [17] and higher levels of internalizing symptoms [18]. Among adolescent girls, the rs2254298 genotype interacts with familiar psychopathology leading to a higher risk of depression and anxiety symptoms [43, 44]. Finally, the rs2740210 polymorphism in the *OXT* gene interacts with early life adversity to predict depressive symptoms [45].

In a cohort of women who recently gave birth to a child, we aimed to investigate whether maternal CM exposure accounts for differences in *OXTR* gene methylation (across regions exon 1 to exon 3) and *OXTR* gene expression. Also, we hypothesized that CM-associated alterations in *OXTR* methylation are modulated by allelic variants of the *OXTR* gene (rs53576 and rs2254298) as well as the *OXT* gene (rs2740210). To address the intergenerational aspects, we first hypothesized that maternal CM experiences are associated with DNAm changes in their newborns. We further assessed associations between the *OXTR*-methylation profile in immune cells collected from mothers and their newborns. By addressing DNAm in immune cells isolated from cord blood from newborns, our study approach allowed investigating the intergenerational perspective minimizing the influence of *post-partum* parenting behaviors. Finally, we tested for G×E interactions between maternal CM and child’s SNP variants to investigate the possible role of rs53576, rs2254298, and rs2740210 on the intergenerational transmission of epigenetic consequences of CM.

## Materials and methods

### Study participants

In total, 533 women who gave birth at the maternity ward of Ulm University Hospital between October 2013 and December 2015 were included in the *My Childhood–Your Childhood* study (See Supplemental Information [SI], section 1 for more detail). After receiving full information about the study, mothers who agreed to participate and did not meet any exclusion criteria (i.e., age under 18 years, insufficient knowledge of the German language, and severe health problems of mother or child during pregnancy or labor), gave written informed consent and provided basic sociodemographic data (*N* = 533). Directly after birth, a maximum of 45 mL fetal blood was collected from the umbilical cord. Within one week following parturition, venous blood was taken from mothers. Blood samples were collected into CPDA-buffered tubes (Sarstedt S-Monovette, Nürmbrecht, Germany) and transported to the laboratory of the Ulm University Department of Clinical & Biological Psychology for the isolation of peripheral blood mononuclear cells (PBMC) from mothers and umbilical blood mononuclear cells (UBMC) from the newborns, respectively. The study protocol and all the procedures were approved by the Ulm University Ethics Committee and was conducted in accordance with the Declaration of Helsinki (2013). All biological material was immediately discarded if mothers did not provide informed consent to study participation.

The German short version of the *Childhood Trauma Questionnaire* (CTQ; [46]) was used to retrospectively assess CM experiences. Using the mild cut-off criteria from Bernstein and Fink (1998; [47]), mothers who reported at least mild CM experiences in at least one CTQ subscale (emotional, physical or sexual abuse, and emotional or physical neglect) were categorized as CM+, and mothers without a history of CM were categorized as CM−. DNAm analyses were conducted in a subset of study participants due to limitations in the availability of biomaterials as described in detail in the SI section 1. The final cohort consisted of 117 mothers (*n* = 59 CM−, and *n* = 58 CM+) and 113 infants. See Table 1 for a summary of the demographic and clinical characteristics of the study cohort. CM+ and CM− mothers did not differ in maternal sociodemographic (i.e. age, origin, academic education, stable relation status) or pregnancy characteristics (i.e. primipara status, smoking status during pregnancy, cesarean section), and none of the characteristics displayed in Table 1 were associated with changes in *OXTR* methylation levels (all *p-*values > 0.05).

**Table 1:** Demographic and biological characteristics.

	Total cohort	CM+	CM−	
	(*N* = 117)	(*n* = 58)	(*n* = 59)	Statistics^a^
Mean age in years (SD)	32.9 (4.4)	32.8 (4.6)	33.1 (4.2)	*t*_*(115)*_ = 0.36, *p* = 0.72
*N* Caucasian maternal ethnicity ^b^ (%)	115 (98.3)	56 (96.6)	59 (100)	*χ*^*2*^_(1)_ = 2.07, *p* = 0.47
*N* academic education^c^ (%)	63 (53.8)	37 (48.3)	31 (59.3)	*χ*^*2*^_(1)_ = 0.52*, p* = 0.47
*N* female sex of infant ^d^ (%)	52 (45.2)	28 (48.3)	24 (40.7)	*χ*^*2*^_(1)_ = 0.68, *p* = 0.41
Mean birth weight (SD) in grams	3387 (497)	3460 (474)	3312 (512)	*t*_*(115)*_ = 1.62, *p* = 0.11
Mean gestational age (SD) in weeks	39.5 (1.4)	39.7 (1.1)	39.3 (1.6)	*W* = 1978.5, *p* = 0.13
*N* cesarean section ^e^ (%)	34 (29.1)	21 (36.2)	13 (23.6)	*χ*^*2*^_(1)_ = 2.03, *p* = 0.15
*N* smokers during pregnancy (%)	11 (8.7)	6 (8.5)	5 (10.3)	*χ*^*2*^_(1)_ = 0, *p* = 0.98
*N* primiparae mothers (%)	58 (49.6)	23 (39.7)	35 (59.3)	*χ*^*2*^_(1)_ = 3.77*, p* = 0.50
*N* in a stable relationship (%)	116 (99.1)	58 (100)	58 (98.3)	*χ*^*2*^_(1)_ = *0*, *p* = 1
***OXTR*** **rs53576 allelic distribution**				
*N* mother A allele carrier (%)	73(62.3)	37 (63.8)	36 (61.0)	*χ*^*2*^_(1)_ = 0.14, *p* = 0.90
*N* children A allele carrier (%)	59 (52.2)	30 (54.5)	29 (50.0)	*χ*^*2*^_(1)_ = 0.87, *p* = 0.77
***OXTR*** **rs2254298 allelic distribution**				
*N* mother A allele carrier (%)	21 (17.9)	10 (17.2)	11 (18.6)	*χ*^*2*^_(1)_ = *0*, *p* = 1
*N* children A allele carrier (%)	24 (21.2)	12 (21.8)	12 (20.7)	*χ*^*2*^_(1)_ = *0*, *p* = 1
***OXT*** **rs2740210 allelic distribution**				
*N* mother C allele carrier (%)	61 (52.1)	28 (48.3)	33 (55.9)	*χ*^*2*^_(1)_ = 0.41, *p* = 0.52
*N* children C allele carrier (%)	66 (58.4)	37 (67.3)	29 (50.0)	*χ*^*2*^_(1)_ = 1.0, *p* = 0.32
**Childhood maltreatment**				
Mean CTQ sum score (SD)	33.6 (10.8)	40.2 (12.1)	27.1 (1.9)	*W* = 103.5, *p* *<* 0.001
Emotional abuse ^f^ (*N* (%))	–	22 (37.9)		
Physical abuse ^f^ (*N* (%))	–	16 (27.6)		
Sexual abuse ^f^ (*N* (%))	–	16 (27.6)		
Emotional neglect ^f^(*N* (%))	–	40 (69.0)		
Physical neglect ^f^ (*N* (%))	–	10 (17.2)		

Group differences calculated with chi-square tests for binomial and t-tests for continuous variables.

*SD* standard deviation, *CM* childhood maltreatment, *CTQ* childhood trauma questionnaire, *CTQ sum score* childhood maltreatment load.

^a^Main effect of the CTQ classification (*t*-tests or *chi-square* tests).

^b^One study participant of Brazilian origin and one of North American origin.

^c^The education information from one CM+ mother was missing.

^d^For gestational and children characteristics, only mother–infant dyads were included: *n*_CM−_=58; *n*_CM+_=55.

^e^Included planned (*n*_CM− _=16, *n*_CM+_=12) and emergency (*n*_CM− _=4, *n*_CM+_=1) forms of cesarean section.

^f^Amount of women with at least mild experiences in the given CTQ subscale.

### DNA methylation analyses

**Fig. 1:**
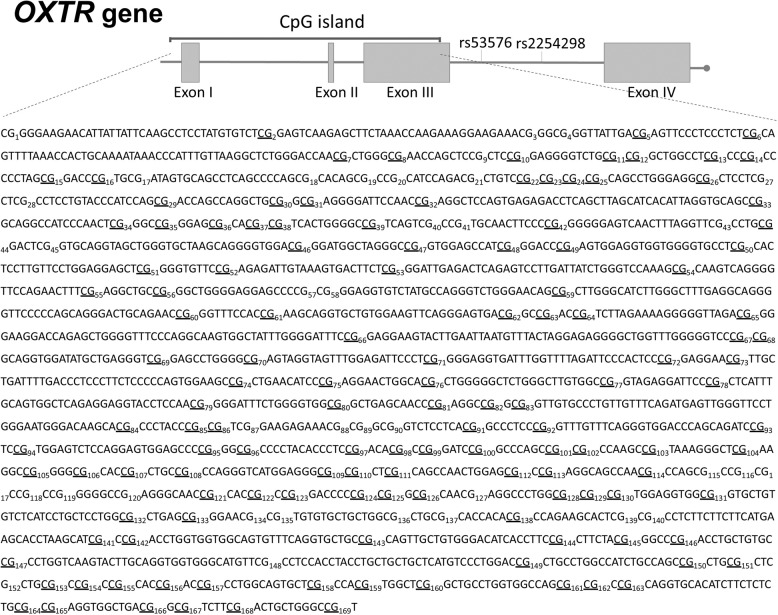
Schematic view of the targeted sequence of the *OXTR* gene. All CpG units located within the targeted sequence (GRCh37/hg19, chr3:8809305-8811438, sequence shown 3' to 5') are numbered consecutively. Underlined are the CpG sites that were included for analyses after data cleaning for the analyses in mothers. Thereafter, 133 CpG sites remained from maternal samples and 142 from children (in contrast to maternal analyses, the CpG 21, CpG 40.41, and CpG 115.116.117.118.119.120 remained for children’s analyses after applying the quality criteria).

For a detailed description of the isolation of (U)PBMC from mothers and newborns and of DNA preparation for mass array please see SI, sections 2 and 3, respectively. The EpiTYPER assay (Sequenom Inc, USA) was used to quantitatively measure the DNAm levels at individual CpG sites. The analyzed region of the *OXTR* gene spans across exons 1, 2, and 3 as well as introns 1 and 2 (Fig. 1). For each CpG unit, the percentage of methylated CpG sites over the sum of methylated and unmethylated CpG sites of the pre-analytical PCR products was used for statistical analyses. As previously reported [9], two quality criteria were applied for data processing: First, CpG units with missing values in more than 30% of the samples were excluded from analyses (Fig. 1). Second, samples with missing values in more than 50% of the CpG units were also excluded, which resulted in the exclusion of one newborn sample only. Thus, the final EpiTYPER dataset consisted of samples from 117 mothers and 112 infants. For statistical analyses of DNAm, the mean percentage of methylation across all remaining CpG sites after data processing was calculated for each individual. Samples were measured blinded to the experimenter.

### Gene-expression analyses

Total RNA was purified from freshly thawed (U)PBMC using the Qiagen RNeasy Kit (QIAGEN, Hilden, Germany) according to the manufacturer’s instructions. The RNA yield was quantified with the Qubit RNA broad range (BR) assay in combination with a Qubit spectrophotometer (Life Technologies). Samples were stored in *RNase* free water (Life Technologies) at −20 °C for a maximum of 7 days prior to cDNA transcription (see SI, section for further details). As previously reported [9] we selected Succinate dehydrogenase complex, subunit A, flavoprotein variant *(SDHA)* and Importin 8 *(IPO8)* as gene expression references. Detailed information about the gene expression analyses and the selection procedure of appropriate house-keeping genes for human (U)PBMC is given in the SI, section 4.

### Allelic discrimination of the SNPs rs53576, rs2254298, and rs2740210

Taqman assays for SNP genotyping are based on the qPCR melting curve approach, which was performed on a QuantStudio 6 platform (Life Technologies, USA). See SI, section 5, for the conditions of thermal cycling. In line with previous research [44, 48–52], we used a dominant model contrasting A-allele carriers (AA/AG genotype) versus G homozygotes (GG) for rs53576 as well as for rs2254298. Regarding *OXT* rs2740210, we dichotomized the AA genotype versus C-allele carriers due to the low frequency of the CC genotype (only 17 mothers were CC carriers). Among the complete cohort, all three SNPs were distributed following the *Hardy Weinberg* equilibrium (rs53576: *χ*^*2*^_(2)_ = 0.014, *p* = 0.90; rs2254298: *χ*^*2*^_(2)_ = 1.14, *p* = 0.29; rs2740210: *χ*^*2*^_(2)_ = 2.77, *p* = 0.10).

### Data pre-processing and statistical analyses

Data pre-processing and statistical analyses were conducted with the statistical software R, version 3.2.3. Shapiro–Wilk tests were used to test the normal distribution of model residuals. Descriptive differences between groups were analyzed using *χ*^*2*^-tests and Student’s *t* tests in case of normally distributed data; otherwise, Mann–Whitney *U*-tests were used. All statistical models included potential confounders for DNAm measurements. The following covariates for the analyses of maternal data were all included in one model: age, percentages of monocytes and lymphocytes in whole blood as separate covariates and days between parturition and isolation of PBMC. For the analyses of infants’ *OXTR* DNAm, sex, smoking during pregnancy, mode of delivery, and gestational age (in weeks) of the infant were included as covariates. For the models testing associations between maternal and infant’s *OXTR* DNAm, maternal age, sex of the infant, and gestational age in weeks were included as covariates. Complete data for all covariates were available for 107 women and 112 infants. None of the covariates showed a main effect on *OXTR* DNAm (all *p-*values *>* 0.05). Maternal *OXTR* DNAm residuals were distributed normally. Here, ANCOVA and multiple linear regressions were used. Newborns’ DNAm and maternal and newborns’ gene expression residuals were not normally distributed. Thus, non-parametric permutation tests [53] of the Student’s *t* tests for group comparisons, and of linear regression models for regressional analyses were used for: (1) all tests including maternal gene expression data, (2) all tests including infant’s DNAm data, (3) all tests including infant’s gene expression data, and (4) interaction tests. Standardized *β* coefficients are reported. For single CpG methylation analyses, the False Discovery Rate (FDR; [54]) was used to correct for multiple testing. All tests were performed two-tailed with a significance threshold of alpha ≤ 0.05 and a CI of 95%.

## Results

### Association of CM experiences and *OXTR* methylation in mothers

**Fig. 2:**
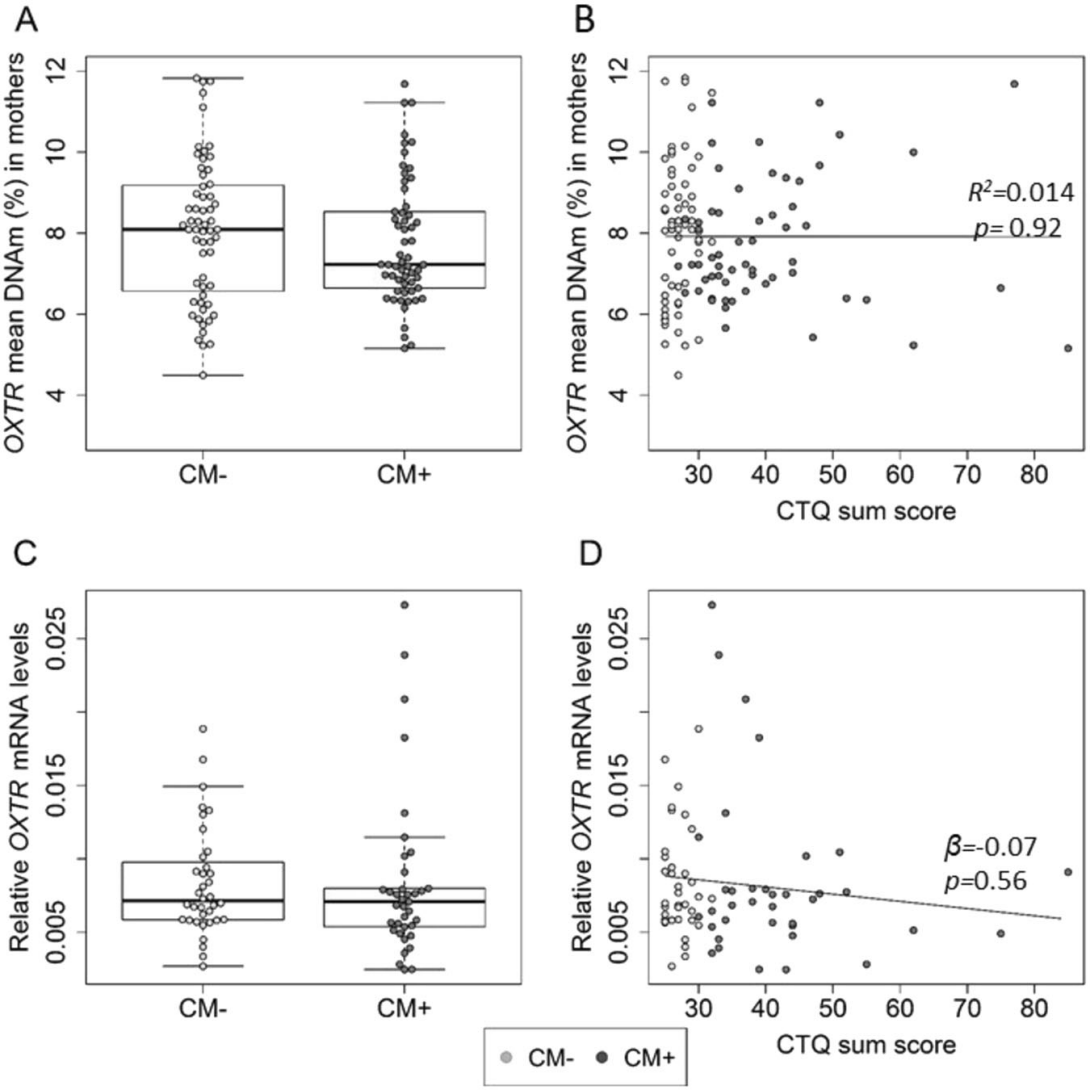
Association between CM and *OXTR* epigenetic regulation in mothers. **A** *OXTR* DNAm did not differ between mothers with childhood maltreatment experiences (CM+; marked with dark points) and without (CM−; marked with light points) *(N* = 107, *F*_*(1,101)*_ = 0.34, *p* = 0.56*)*. **B** The severity of CM experiences (measured as the CTQ sum score) did not correlate with maternal *OXTR* DNAm (*R*^*2*^ = 0.014, *p* = 0.92). **C**, **D** The relative *OXTR-*gene expression did not differ between CM+ and CM− mothers (*N* = 73, *β* = −0.01, *p* = 0.93) and did not correlate with the severity of CM (*N* = 73, *β* = −0.07, *p* = 0.56). Whiskers indicate variability outside the upper and lower quartiles.

Across the targeted sequence of the *OXTR*, mean DNAm did not differ significantly between CM+ (*M* ± SD = 7.7% ± 1.5%) and CM− (*M* ± SD = 8.1% ± 1.8%; *N* = 107, *F*_(1,101)_ = 0.34, *p* = 0.56; Fig. 2A) and did not correlate with CM load (*R*^*2*^ = 0.014, *p* = 0.92; Fig. 2B), represented by the CTQ sum score. Finally, none of the CTQ subscales showed associations with *OXTR* DNA methylation (all *p-*values > 0.05.

For analyses on a single CpG level, we used permutation analysis of the linear regression between CM and DNA methylation of all CpG units (a total of 76). Fifteen CpG units were significantly associated with CM status (see Table S1 in SI for results). After adjusting for multiple comparisons by FDR, 4 CpG remained significant: CpG 169, CpG 2, CpG 5, and CpG 6. Methylation levels of the CpG 169 were significantly higher in CM+ compared to CM− (*β* = 0.29, *p*_adj_ = 0.04). CpG 2 (*β* = −0.41, *p*_adj _< 0.0001), CpG 5 (*β* = −0.34, *p*_adj_ = 0.01), and CpG 6 (*β* = −0.40, *p*_adj _< 0.0001) was significantly less methylated in CM+ compared to CM−. There were no associations between methylation levels of the targeted CpG units and CM load (all *p*_adj _> 0.05). See SI, Table S1 for detailed information on single CpG results.

*OXTR* was not differentially expressed in PBMC depending on CM status or CM load (Figs. 2C, D). *OXTR* methylation and *OXTR* gene expression were not significantly associated in the mothers (*N* = 73, *β* = 0.01, *p* = 0.92).

### G×E interactions on *OXTR* methylation and expression among mothers

**Fig. 3:**
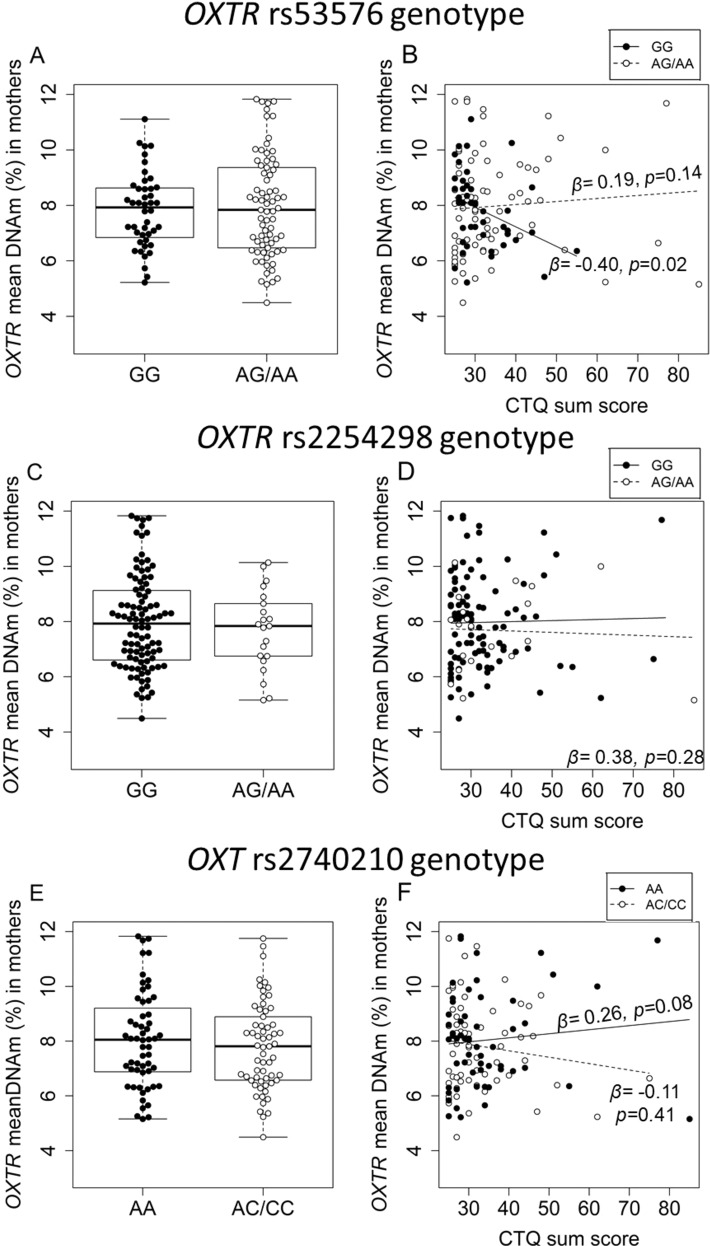
Interaction of rs53576, rs2254298, and rs2740210 with childhood maltreatment (CM) load on maternal *OXTR* DNAm among mothers. **A** *OXTR* mean DNAm did not differ significantly between mothers with the GG genotype for the *OXTR* rs53576 (*n* = 41) and A-allele carrier mothers (*n* = 66) (*β* = 0.03, *p* = 0.78). **B** The *OXTR* rs53576 genotype modulated *OXTR*-methylation levels in interaction with the severity of CM experiences (*N* = 107, *β* = *1.24, p* = 0.01). Further analyses revealed that only mothers with the GG genotype of the rs53576 showed a negative association between the CTQ sum score and *OXTR* methylation (*n* = 41, *β* = −0.40*, p* = 0.02), while A-allele carriers did not (*n* = 66, *β* = 0.19, *p* = 0.14). **C** The *OXTR* DNAm did not differ between mothers carrying at least one A allele of the *OXTR* rs2254298 polymorphism (*n* = 18) and GG-homozygous mothers (*n* = 89, *β* = −0.04, *p* = 0.70). **D** The rs2254298 genotype did not interact with the severity of CM experiences in predicting maternal *OXTR* DNAm (*N* = 107, *β* = 0.38*, p* = 0.28). **E** Mothers carrying a C-allele of the *OXT*-rs2740210 SNP (*N* = 56) did not differ from AA-homozygous mothers (*n* = 51, *β* = −0.05; *p* = 0.59) with regard to *OXTR* methylation. **F** However, the rs2740210 modulated DNAm levels in interaction with CM severity (measured as the CTQ sum score) (*N* = 107, *β* = −0.84*, p* = 0.02). While AA-homozygous women exhibited a trend of higher *OXTR* DNAm with higher CM severity (*n* = 51; *β* = *0.26, p* = 0.08), no such association was observed among women carrying a C allele (*n* = 56; *β* = −0.11, *p* = 0.41).

Even though none of the SNP genotypes exerted main effects on *OXTR* DNAm (Fig. 3A, C, E), the *OXTR* rs53576 genotype interacted with CM load in modulating the mean *OXTR* methylation levels (*β* = 1.24*, p* = 0.01; Fig. 3B). Subsequent allele-specific analyses revealed that only mothers with the rs53576 GG genotype show a negative association between CM load and *OXTR* mean methylation (*β* = −0.40*, p* = 0.02), but not A-allele carriers (*β* = 0.19, *p* = 0.14). There was also an interaction between *OXT* rs2740210 genotype and CM load (*β* = −0.84*, p* = 0.02) that modulated *OXTR* methylation levels (Fig. 3F), but not between the *OXTR* rs2254298 and CM load (*β* = 0.38*, p* = 0.28; Fig. 3D). Only women with the AA genotype (rs2740210) showed a tendency for a positive association between the CM load and the *OXTR* DNAm (*β* = 0.26, *p* = 0.08).

*OXTR* gene expression was not modulated by the interaction between any of the SNPs and CM status (*N* = 67; CM ⨯ rs53576: *β* = −0.15, *p* = 0.52; CM ⨯ rs2254298: *β* = −0.20, *p* = 0.19; CM ⨯ rs2740210: *β* = 0.33, *p* = 0.11).

### Effects of maternal CM experiences on newborns’ *OXTR* methylation and gene expression

**Fig. 4:**
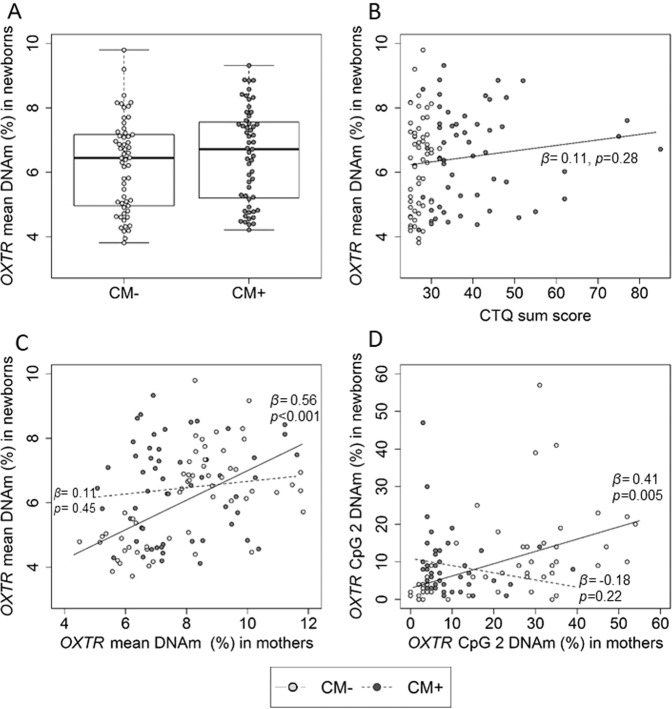
Effects of maternal childhood maltreatment (CM) experiences on infant’s *OXTR* DNAm levels. The severity of maternal CM experiences was not associated with alterations in the DNA methylation among newborns. **A** DNAm did not differ between infants from CM+ (*n* = 55) and CM− mothers (*n* = 57) (*β* = 0.07, *p* = 0.45). **B** The severity of maternal CM experiences was not associated with newborns’ *OXTR* DNAm (*β* = 0.11, *p* = 0.28). **C** Mean *OXTR* DNAm of mothers and their offspring correlated positively (*N* = 112, *β* = 0.34, *p* < 0.001). There was an interaction effect between maternal *OXTR* methylation and maternal CM on newborn’s *OXTR* methylation (*N* = 112, *β* = −0.95, *p* = 0.03). Further analyses confirmed a positive association between maternal and newborns *OXTR* methylation specifically for CM− mothers (*β* = 0.56, *p* *<* 0.001) but not CM+ mothers (*β* = 0.11, *p* = 0.45). **D** At *OXTR* CpG 2, maternal DNAm was linked to infants’ DNAm depending on maternal CM: Maternal and infant’s CpG 2 DNAm levels were positively correlated only among dyads with CM− mothers (*β* = 0.41, *p* = 0.005), but not among dyads with CM+ mothers (*β* = −0.18, *p* = 0.22).

There were no differences in *OXTR* DNAm between boys or girls (*N* = 112; *W* = 1427*; p* = 0.46). *OXTR* DNAm levels did not differ (*N* = 112, *β* = 0.07, *p* = 0.45; Fig.4A) between infants of CM+ mothers (*M* ± SD = 6.5 ± 1.5%) infants of CM− mothers (*M* ± SD = 6.2 ± 1.4%). Among the complete cohort, the severity of maternal CM experiences (CM load) was not associated with children *OXTR* methylation levels (*N* = 112*, β* = 0.11, *p* = 0.28, Fig. 4B), also independently of the sex. Single CpG unit analyses showed that newborns’ DNAm levels at specific CpG sites were not associated with maternal CM experiences for any of the analyzed CpGs (all *p-*values > 0.05).

Overall mean *OXTR* DNAm levels of mothers and their offspring were positively correlated (*N* = 112, *β* = 0.34, *p* *<* 0.001; Fig. 4C). Permutation tests of the linear regression model showed an interaction effect between *OXTR* methylation levels among mothers and their CM status on infant’s *OXTR* methylation levels (*N* = 112, *β* = −0.95, *p* = 0.03). Further analyses confirmed that only CM− mother–infant dyads showed a positive association between their mean *OXTR* DNAm levels (*n* = 57, *β* = 0.56, *p* *<* 0.001), but not CM+ mother–infant dyads (*n* = 55, *β* = 0.11, *p* = 0.45) (Fig. 4C). When we stratified by sex, again only CM− dyads showed the mother–infant positive association between their mean *OXTR* DNAm levels (Girls: CM− dyads: *n* = 24, *β* = 0.45, *p* = 0.02; CM+ dyads: *n* = 28, *β* = 0.08, *p* = 0.76/Boys: CM− dyads: *n* = 33, *β* = 0.71, *p* *<* 0.001; CM+ dyads: *n* = 27, *β* = 0.10, *p* = 0.43).

We further analyzed the associations between the *OXTR* DNAm of mothers and their newborns on a single CpG unit level. To this end, we correlated maternal and infant’s DNAm only for those CpG candidates for which an effect of CM exposure was found among mothers (CpG 2, CpG 5, CpG 6, and CpG 169). Across the complete cohort, CpG 2 DNAm levels were positively associated between mothers and infants (*N* = 98, *β* = 0.27, *p* = 0.008). Further analyses showed an interaction between maternal CpG 2 DNAm and the CM status on the infant´s CpG 2 DNAm (*N* = 98, *β* = −0.36, *p* = 0.02; Fig. 4D).

Relative *OXTR* RNA levels did not differ in the UBMC of infants of CM+ mothers compared to those of CM− mothers (*N* = 38, *β* = 0.01, *p* = 0.96) and were not associated with maternal CM load (*N* = 38, *β* = 0.06, *p* = 0.76). *OXTR* methylation and *OXTR* gene expression were not significantly associated in infants (*N* = 38, *β* = 0.12, *p* = 0.51).

### G×E interactions on *OXTR* methylation and expression among infants

None of the infants’ SNPs interacted with maternal CM status on infants’ methylation levels (*N* = 112; rs53576: *β* = −0.12, *p* = 0.46; rs2254298: *β* = −0.17, *p* = 0.23; rs2740210: *β* = −0.19; *p* = 0.24), nor did maternal SNP variants (all *p*-values > 0.05). Moreover, maternal and infants’ genotypes of the analyzed SNPs did not modulate the association between infants’ and maternal *OXTR* DNAm levels (all *p-*values > 0.05). Finally, *OXTR* gene expression did not depend on the interaction between the analyzed SNPs and maternal CM experiences (*N* = 34; all *p*-values > 0.05).

## Discussion

This study aimed at investigating the intra- and intergenerational effects of CM on the epigenetic regulation of the *OXTR* gene. The most important finding is that maternal and newborns mean *OXTR* DNAm levels were positively associated in mothers without a history of CM, but not in mothers with CM experiences. While CM load had no main effect on maternal mean DNAm of the *OXTR* gene, we observed a CpG-specific effect of CM on the hypomethylation of CpG 169 and hypermethylation of CpG 2, CpG 5, and CpG 6 within the *OXTR* gene of mothers. Finally, *OXTR* rs53576 and *OXT* rs2740210 polymorphisms interacted with the severity of CM experiences on *OXTR* mean DNAm in mothers: only women with the GG genotype at rs53576 showed a negative correlation between the CTQ sum score and *OXTR* methylation, and only women with at least one C-allele in the rs2740210 SNP showed a tendency for a positive association between CM load and *OXTR* methylation.

Our results suggest that the intergenerational transmission of the epigenetic regulation of the oxytocinergic system differs in mothers with CM experiences and their newborns when compared to controls, independently of the sex of the offspring. These results can be interpreted from several perspectives: From an evolutionary neo-Lamarckian point of view, our results suggest that maternal epigenetic adaptations might only be perpetuated across CM− dyads, and not across CM+. In CM− dyads, this transmission may prepare the next generation to deal with stress. However, in CM+ dyads it might not be evolutionary adaptive to transmit the maternal adaptations, which were presumably acquired to deal with severe, detrimental experiences and thus do not provide evolutionary fitness under normal circumstances [55]. Accordingly, a recent meta-analysis discusses the role of natural selection in the developmental programming of oxytocinergic after early life adversity and CM [39]. From a physiological perspective, given the modulating role of the OXT-system on the immune system, and our results in immune cells, one could speculate that this is one of the multiple mechanisms explaining why offspring from CM+ mothers are at increased risk for psychological and physical health [56, 57]. In line with our results, previous literature highlighted the regulatory role of the oxytocinergic system on the impact of stress across generations. For example, the total number of maternal critical life events up to 2 years before the second trimester predicted *OXTR* DNAm of newborns’ cord blood cells [36]. Moreover, it has been suggested that the OXT pathway starts preparing for parenting behavior already during pregnancy [58]. With regards to the effects on single CpG sites, of special interest is the CpG 2, as its methylation correlated between mothers and their infants only in the CM-free dyads. Regarding the role of the *SNPs* rs53576, rs2254298, and rs2740210, in our study the CM-associated changes on the comparability of *OXTR* DNAm between mothers and infants occurred independently of the maternal or newborn’s genotype. However, previous findings suggested that maternal transmission of psychopathology depends on the *OXTR* rs2254298 genotype of the daughters [44].

Among mothers, we observed associations of CM with altered DNAm level in four CpG units but not on the mean DNAm of *OXTR*, suggesting that CM-associated changes of *OXTR* methylation are CpG-specific. CpG 169 methylation was higher among CM+ mothers, while the methylation of CpG 2, CpG 5, and CpG 6 was lower among CM+ mothers compared to the CM− group. These results showing a CpG-specific methylation pattern indicate a complex epigenetic regulation of the *OXTR* gene. Previous studies have associated childhood adversity with higher *OXTR* methylation [29, 59, 60]. However, the correlational directions of the environment on gene methylation have been inconsistent [61]. This inconsistency might result from (i) differences in cohorts, (ii) differences in CM assessment and classification, and (iii) differences in analytical methods used to measure DNAm (e.g. MassARRAY vs. Illumina).

Previous studies suggested a higher sensitivity to the effects of early life stress exposure in GG carriers for the rs53576 *OXTR* genotype [34, 62, 63]. We found that only GG-homozygous women showed lowered *OXTR* DNAm with increased CM load. This rs53576-dependent reduction of the DNAm might reflect the differential susceptibility reported in the literature [64], and the body’s attempt to regulate *OXTR* expression during the post-partum period when parenting behavior becomes especially important. Regarding the rs2740210 SNP, our results complement previous genetic evidence about the predictive role of rs2740210 on individual mothering behavior [45, 65]. We provide the first epigenetic evidence that the level of *OXTR* methylation in immune cells is regulated by an interaction of rs2740210 *×* CM exposure. Yet, these results have to be interpreted with caution due to the right-skewed distribution of the data and the low prevalence of severely traumatized individuals in the sample that leads to heteroscedasticity problems. Although we tried to counter this problem by using robust permutation analyses of the linear regression models, the strengths of the association of these findings are relatively weak. Finally, we did not find any associations between rs2254298 and CM-associated *OXTR* regulation, which is in line with a study that found that rs53576 and rs2254298 do not modulate the associations between CM and depression or anxiety in adulthood [66]. In sum, our findings indicate differential epigenetic adaptation of *OXTR*-DNAm levels in immune cells to CM exposure depending on the *OXTR* rs53576, and presumably *OXT* rs2740210, genotypes.

In this study, the observed methylation changes were not associated with alterations in gene expression. In contrast to our findings, one study using luciferase reporter gene expression assays showed that higher methylation within the same CpG island analyzed in our study resulted in lowered *OXTR* gene expression [67]. It is important to note that the degree of suppression by DNA methylation was tissue-dependent [67]. Using two house-keeping genes as a reference we here show that, at least in PBMC, alterations in the mean or site-specific DNAm of *OXTR* might not consequently lead to alterations of its gene expression. In a recent study, we found that CM-affected women showed reduced OXTR protein density in PBMC compared to women without CM [24]. We thus speculate that the *OXTR* is exposed to an additional level of genetic regulation in immune cells. Indeed, *OXTR* gene expression is activated via the inflammation regulator NF-kB in human macrophages [68], which might protect immune cells under distress in the presence of external stressors such as CM. In line with this hypothesis, previous studies suggested immune-modulating effects of OXT [69] and higher peripheral OXT levels were associated with a reduction of oxygen consumption of immune cells and shortened telomere length [22, 23]. Therefore, future studies should investigate the potential effects of OXT regulation on stress-associated inflammatory disease outcomes, and extend our results to cell sub-population levels to elucidate the immunological implications of our observations.

Some limitations need to be taken into account when interpreting the findings. First, as sex-specific effects on the *OXTR* regulation after early life adversities have been reported [29, 60], future studies should also investigate father-newborn dyads. Second, the data were generated with PBMC and cannot be generalized to other tissues such as neurons. While other studies used buccal cells, we used PBMC because they are physiologically more comparable to neurons than any other peripheral tissue. Accordingly, a previous genome-wide association study (GWAS) showed similar patterns of *OXTR* regulation in immune cells and brain tissue [70]. Third, one significant difference between maternal venous and newborns’ cord blood cells is the presence of CD34+ (embryonic stem cells) in umbilical cord blood. As a consequence, the epigenetic signature from both groups might reflect the different cellular composition. Future studies investigating methylation change on the isolated blood-cell subsets, e.g. T and B cells and CD34+ cells are warranted. Some limitations are related to the special nature of our cohort, which included only puerperal mothers. Pregnancy, and especially delivery, can increase physiological oxytocin and cortisol levels, which usually peak at the end of the third trimester [71–73]. Moreover, we did not address breastfeeding status, lacerations, or whether mothers received exogenous oxytocin during delivery. Delivery wounds, lack of sleep, and perinatal hormonal fluctuations may impact oxytocinergic physiological regulation. However, whether such post-partum factors directly affect methylation of *OXTR* itself remains uninvestigated. Our results do not show associations between *OXTR* methylation and other perinatal factors like being primipara mother and type of delivery. With regards to OXT infusions, so far there are inconsistent findings on whether OXT can transfuse through the placenta [74, 75]. Future studies are warranted to investigate implications of breastfeeding, lacerations, postnatal inflammatory status, and OXT infusions during delivery on *OXTR* methylation. Lastly, our cohort consisted only of healthy women who reported relatively low levels of CM load, which might mask some effects of more severe CM experiences and associated clinical outcomes on the *OXTR* methylation. Besides negative CM experiences, future studies should also consider alternative scales for CM assessments and especially the protective role of positive parenting experiences.

In conclusion, this exploratory study showed rather specific effects of CM exposure on the *OXTR* modulation in immune cells of mother–newborn dyads. Most importantly, we found indications that the maternal CM status might interfere with the biological inheritance of the *OXTR* regulation from the mother to the child—at least on the level of immune cells. Regarding G×E interaction, our results suggest a protective effect of the GG genotype for the rs53576 SNP. We further provide first evidence for a potential modulating role of the *OXT* rs2740210 polymorphism on the regulation of the oxytocinergic system in women with a history of CM experiences, but these results need future replication. Our findings shed light on the complex regulation of the *OXTR* gene in the context of CM and highlight the importance of the oxytocinergic system as a potential candidate to further investigate the psychobiological consequences of CM and how maternal CM experiences might disrupt the normal transmission of *OXTR* from generation to generation.

## Supplementary information

Supplemental Material_Ramo et al_CM and OXTR
