# Supplementary material for: Associations between childhood maltreatment and DNA methylation of the oxytocin receptor gene in immune cells of mother–newborn dyads
Source: Transl Psychiatry. 2021 Sep 1;11:449. doi: 10.1038/s41398-021-01546-w (PMC8410844; doi:10.1038/s41398-021-01546-w)
Supplement: Supplementary file 1 — Supplemental Material_Ramo et al_CM and OXTR [file 41398_2021_1546_MOESM1_ESM.docx]

**Associations between childhood maltreatment and DNA methylation of the oxytocin receptor gene in immune cells of mother-newborn dyads**

Laura Ramo-Fernández^1*^, Anja M.Gumpp^1^, Christina Boeck^1^, Sabrina Krause^2^, Alexandra M. Bach^1^, Christiane Waller^2,3^, Iris-Tatjana Kolassa^1^, Alexander Karabatsiakis^1*^

**Supplemental Information**

^1^ Clinical & Biological Psychology, Institute of Psychology and Education, Ulm University, Ulm, Germany

^2^ Psychosomatic Medicine and Psychotherapy, University Hospital Ulm, Ulm, Germany

^3^ Department of Psychosomatics and Psychotherapeutic Medicine, Paracelsus Medical Private University of Nuremberg, Nuremberg, Germany

^4^ Department of Clinical Psychology II, Institute of Psychology, University of Innsbruck, Innsbruck, Austria

*Corresponding authors:

Laura Ramo-Fernández

Clinical & Biological Psychology

Institute of Psychology and Education

Ulm University

Albert-Einstein-Allee 47, 89081 Ulm, Germany

E-mail: laura.ramo.fernandez@gmail.com

Alexander Karabatsiakis

Department of Clinical Psychology II

Institute of Psychology

University of Innsbruck,

Bruno-Sander-Haus, Innrain 52f, 6020 Innsbruck, Austria

Alexander.Karabatsiakis@uibk.ac.at

1. *Study participants included for epigenetic and gene expression analyses*

A total of 5426 women who gave birth in the maternity ward of Ulm University between October 2013 and December 2015 were asked for participation in the project *My Childhood-Your Childhood*. From them, 533 mother-infant dyads were recruited in the study *My Childhood – Your Childhood*. After screening, *n*=58 dyads with a maternal history of CM (CM+) and blood samples available from both, mother and newborn, for analytical processing were included for epigenetic analyses (for a detailed description see Ramo *et al.* 2019; 1). A cohort of *n*=59 dyads without a history of CM (CM-) were selected as controls for epigenetic analyses. The two groups (were matched for maternal age, gestational week at the time of birth, birth weight, and sex of the infant. The number of infants in the dataset was reduced by 1 for the CM- group and 2 for the CM+ group because of twin birth, which was rated as an exclusion criterion afterwards, dropping the final cohort to 114 infants. One infant blood sample (CM+ group) had to be excluded due to technical failure during dense gradient isolation. Thus, the final sample size included 117 mothers (*n=*58 CM+ and *n*=59 CM-), 113 infants *(n*=55 CM+ and *n*=58 CM-), and 113 mother-infant-dyads. One 2.7 ml EDTA-buffered blood collection tube (Sarstedt S-Monovette, Nürmbrecht, Germany) was filled with venous blood to assess the percentages of monocytes and lymphocytes in the Department of Clinical Chemistry at the Ulm University Clinic. Mothers who were included in the epigenetic sub-study of the *My childhood –Your childhood* project (*N=*117) did not differ from mothers from the larger sample who were not included (*N*=416) in terms of maternal chronological age, gender and body weight of their newborns, ethnicity, cigarette smoking during pregnancy, chronic illnesses, lifetime psychological diagnosis or medication intake during pregnancy (all p-values >.05).

1. *Isolation and cryopreservation of immune cells*

Isolation of (U)PBMC from mothers and infants was performed using Ficoll-Hypaque density gradient centrifugation (GE Healthcare, UK) and Leukosep tubes (Greiner Bio-One, Germany) following the manufacturer’s protocol. After resuspension into pre-chilled (4° C) cryopreservation medium (dimethyl sulfoxide: Sigma-Aldrich, USA; fetal calf serum: Sigma-Aldrich; dilution: 1:10 v/v), cell aliquots were stored at -80° C and until DNA isolation. DNA from (U)PBMC was isolated using the MagNA Pure 96 system (Roche, Switzerland) for epigenetic and SNP genotyping analyses.

1. *DNA preparation for mass array analyses and genotyping analyses*

The concentration of isolated DNA was quantified using Qubit DNA Broad Range kits and a Qubit spectrophotometer (Life Technologies, USA). After lyophilization in a CentriVap concentrator (Labconco Corp, MO, USA), DNA was resuspended in DNAse-free water (Life Technologies, USA) to obtain a final concentration of 40 ng dsDNA per µl. DNA aliquots were stored at -20° C and provided frozen to Varionostic GmbH (Ulm, Germany) for MassARRAY analyses.

1. *Gene expression analyses and selection of housekeeping reference genes*

For a certain number of participants, the total amount of isolated PBMC/UBMC was not sufficient to isolate the appropriate amount of RNA needed for the gene expression analyses. Thus, their samples (*n*=50 mothers, *n*=79 newborns) were excluded from the processing for the isolation of RNA. As a result, RNA was isolated only from 67 mothers and 34 infants using the Qiagen RNeasy Kit (QIAGEN, Hilden, Germany). The RNA yielded was quantified with a Qubit spectrophotometer (Life Technologies) and stored in RNase free water (Life Technologies) at -20ºC. Up to seven days after RNA isolation, cDNA transcription was performed using the high-capacity cDNA reverse transcription kit following the manufacturer’s instructions (Thermo Fischer Scientific, Germany). Gene expression analyses for *OXTR* were performed on a QuantStudio 6 qPCR platform (Life Technologies, USA) using the appropriate Taqman gene expression array (Hs00168573_m1; Thermo Fischer Scientific). Out of a total of five candidate genes (SDHA, IPO8, 18S, TBP, RPL13A) that were reported in literature to be constantly expressed in human PBMC (2) and not influenced by CM, two internal controls were selected as reference genes: succinate dehydrogenase complex, subunit A (*SDHA*) and importin 8 (*IPO8*). Using the program NormFinder (3), an algorithm-based test that identifies the optimal normalization gene among a set of candidates, we ranked the candidate genes according to their expression stability in the given sample set and experimental design. Based on this algorithm, the combination of *IPO8* and *SDHA* generated the strongest stability and were thus selected as reference genes for the gene expression analyses in the given study. Group comparisons revealed that both housekeeping genes were equally expressed between CM- and CM+ mothers (*t(SDHA)* _(1,64)_=-0.93, *p*=.35; *t(IPO8)*_(1, 66)_=0.13, *p*=.90). For Taqman-based real-time PCR, triplicates of 20 ng cDNA in a total volume of 20 µl were used. The average Ct of the triplicates was calculated and the relative mRNA levels of OXTR were defined with the 2^-ΔCT^ equation, with ∆Ct = (mean Ct of the target) – (geometric mean of the Ct of the reference genes SDHA and IPO8). The resulting fold-change values – an estimate of relative mRNA expression levels – were used for statistical analyses.

1. *OXTR rs53576, OXTR rs2254298, and OXT rs274010 genotyping conditions*

In total, three SNPs were genotyped, namely rs53576 (C___3290335_20,) and rs2254298 (C___2269915_10) within the *OXTR* gene, and rs2740210 (C___16061225_10) within the *OXT* gene*.* Thermal cycling conditions started with 10 min of enzyme activation at 95° C followed by 40 cycles with 15 sec at 95° C, and 1 min at 60° C. Positive (DNA with known genotype) and negative (absence of DNA) controls were used for additional quality control. The minor allele frequency (MAF) was 38.4% for the rs53576 SNP (A allele), 8.9% for the rs2254298 (A allele), and 33.3% for the rs274010 (C allele) within the mothers and 33.6%, 11.9%, and 34.5% respectively within their infants.

**Reference list from Supplementary Information**

1. Ramo-Fernández, L., et al*.* The effects of childhood maltreatment on epigenetic regulation of stress-response associated genes: an intergenerational approach. *Scientific Reports.*  **9**, 983 (2019).
2. Ledderose, C., Heyn, J., Limbeck, E., & Kreth, S. Selection of reliable reference genes for quantitative real-time PCR in human T cells and neutrophils. *BMC Res. Notes* **4,** 427 (2011).
3. Andersen, C. L., Jensen, J. L., & Ørntoft, T. F. Normalization of real-time quantitative reverse transcription-PCR data: a model-based variance estimation approach to identify genes suited for normalization, applied to bladder and colon cancer data sets. *Cancer Res*. **64**, 5245-5250 (2004).

| **Table S1. Specific CpG units analyses in mothers** | | | |  |  | |  |  |  |  |  |
| --- | --- | --- | --- | --- | --- | --- | --- | --- | --- | --- | --- |
|  | **Group-wise analyses** | | | | | | |  | **Association with CTQ sum score** | | |
|  |  | **CM-** | **CM+** |  |  | |  |  |  |  |  |
| **CpG unit** | ***N^a^*** | **Mean % methylation (SD)** | **Mean % methylation (SD)** | **Statistics^b^** | **not adjusted *p*-value** | | ***p-* value BH adjusted ^d^** |  | **Statistics** | **not adjusted *p*-value** | ***p-* value BH adjusted** |
| **CpG 2** | 114 | 23 (19) | 10 (9) | -0.41 | < 0.001 | | **<0.0001** |  | -0.15 | 0.13 | 0.39 |
| **CpG 5** | 113 | 3 (2) | 2 (1) | -0.34 | < 0.001 | | **0.01** |  | -0.20 | 0.05 | 0.24 |
| **CpG 6** | 114 | 8 (9) | 3 (3) | -0.40 | < 0.001 | | **<0.0001** |  | -0.21 | 0.04 | 0.24 |
| CpG 7 | 114 | 3 (1) | 3 (2) | -0.02 | 0.81 | | 0.89 |  | -0.08 | 0.41 | 0.80 |
| CpG 8 | 114 | 3 (2) | 3 (2) | 0.01 | 0.90 | | 0.93 |  | 0.05 | 0.63 | 0.84 |
| CpG 10.26 | 114 | 6 () | 6 (2) | 0.01 | 0.95 | | 0.96 |  | 0.09 | 0.39 | 0.80 |
| CpG 11.12 | 114 | 2 (2) | 3 (1) | 0.04 | 0.73 | | 0.89 |  | -0.05 | 0.60 | 0.84 |
| CpG 13.14 | 110 | 5 (6) | 4 (3) | -0.17 | 0.08 | | 0.29 |  | -0.07 | 0.45 | 0.80 |
| CpG 15.16 | 113 | 3 (2) | 3 (2) | -0.09 | 0.37 | | 0.70 |  | -0.02 | 0.84 | 0.90 |
| CpG 22.23.24.25 | 114 | 3 (1) | 4 (2) | 0.22 | 0.03 | | 0.16 |  | 0.07 | 0.45 | 0.80 |
| CpG 29 | 114 | 2 (1) | 2 (1) | -0.25 | 0.01 | | 0.11 |  | -0.03 | 0.77 | 0.88 |
| CpG 30.31 | 113 | 8 (6) | 7 (4) | -0.02 | 0.85 | | 0.89 |  | 0.06 | 0.51 | 0.80 |
| CpG 32 | 113 | 5 (6) | 3 (7) | -0.12 | 0.28 | | 0.65 |  | -0.04 | 0.65 | 0.84 |
| CpG 33 | 113 | 4 (2) | 3 (1) | -0.09 | 0.35 | | 0.70 |  | 0.07 | 0.52 | 0.80 |
| CpG 34.35.36.37.38 | 105 | 12 (3) | 12 (3) | -0.04 | 0.70 | | 0.89 |  | 0.05 | 0.65 | 0.84 |
| CpG 39 | 115 | 15 (6) | 12 (5) | -0.18 | 0.06 | | 0.27 |  | -0.20 | 0.04 | 0.24 |
| CpG 42.54 | 113 | 20 (6) | 20 (5) | 0.05 | 0.64 | | 0.89 |  | 0.10 | 0.30 | 0.68 |
| CpG 44.55 | 115 | 11 (3) | 10 (3) | -0.13 | 0.19 | | 0.54 |  | 0.07 | 0.50 | 0.80 |
| CpG 46 | 115 | 12 (4) | 10 (3) | 0.22 | 0.02 | | 0.15 |  | -0.18 | 0.07 | 0.27 |
| CpG 48.49 | 110 | 10 (3) | 11 (3) | F(1.95)=5.91 | 0.02 | | 0.14 |  | r=0.11 | 0.15 | 0.43 |
| CpG 47.52 | 115 | 29 (1) | 28 (7) | -0.06 | 0.57 | | 0.88 |  | 0.02 | 0.82 | 0.90 |
| CpG 50 | 93 | 17 (5) | 15 (5) | F(1.78)=0.87 | 0.35 | | 0.70 |  | *r*=0.04 | 0.72 | 0.87 |
| CpG 51.53 | 114 | 41 (13) | 39 (10) | F(1.99)=0.12 | 0.73 | | 0.89 |  | *r*=0.07 | 0.50 | 0.80 |
| CpG 56 | 115 | 14 (5) | 13 (5) | 0.02 | 0.84 | | 0.89 |  | 0.03 | 0.74 | 0.87 |
| CpG 59 | 112 | 16 (7) | 16 (6) | F(1.97)=.01 | 0.72 | | 0.89 |  | r=0.07 | 0.47 | 0.80 |
| CpG 60 | 115 | 4 (2) | 4 (3) | 0.01 | 0.96 | | 0.96 |  | 0.25 | 0.01 | 0.14 |
| CpG 61 | 115 | 6 (4) | 6 (6) | 0.03 | 0.79 | | 0.89 |  | 0.27 | 0.01 | 0.13 |
| CpG 62.63.64 | 115 | 3 (2) | 3 (2) | 0.06 | 0.52 | | 0.86 |  | 0.21 | 0.04 | 0.24 |
| CpG 65 | 102 | 5 (2) | 5 (3) | 0.05 | 0.60 | | 0.89 |  | 0.29 | 0.01 | 0.13 |
| CpG 66 | 115 | 13 (6) | 13 (8) | 0.04 | 0.70 | | 0.89 |  | 0.16 | 0.11 | 0.34 |
| CpG 67.68 | 114 | 4 (3) | 5 (6) | 0.14 | 0.16 | | 0.48 |  | 0.23 | 0.02 | 0.19 |
| CpG 69.76 | 115 | 12 (5) | 13 (5) | 0.16 | 0.12 | | 0.41 |  | 0.31 | 0.00 | 0.13 |
| CpG 70.71 | 115 | 12 (4) | 12 (6) | 0.02 | 0.84 | | 0.89 |  | 0.27 | 0.01 | 0.13 |
| CpG 72.73 | 115 | 10 (3) | 11 (4) | 0.21 | 0.03 | | 0.17 |  | 0.19 | 0.05 | 0.24 |
| CpG 74 | 115 | 7 (3) | 8 (4) | 0.02 | 0.85 | | 0.89 |  | 0.04 | 0.65 | 0.84 |
| CpG 75 | 115 | 5 (2) | 4 (3) | F(1.99)=0.679 | 0.41 | | 0.75 |  | r=0.06 | 0.52 | 0.80 |
| CpG 78 | 109 | 3 (5) | 5 (8) | 0.17 | 0.08 | | 0.29 |  | 0.14 | 0.17 | 0.47 |
| CpG 79 | 115 | 3 (2) | 3 (2) | -0.14 | 0.16 | | 0.48 |  | -0.03 | 0.74 | 0.87 |
| CpG 80.77 | 115 | 9 (4) | 8 (4) | -0.05 | 0.61 | | 0.89 |  | 0.05 | 0.60 | 0.84 |
| CpG 81.82.83 | 99 | 5 (2) | 5 (2) | -0.12 | 0.25 | | 0.64 |  | -0.06 | 0.57 | 0.84 |
| CpG 84 | 113 | 3 (3) | 3 (3) | 0.03 | 0.78 | | 0.89 |  | 0.19 | 0.05 | 0.24 |
| CpG 85.86 | 115 | 2 (2) | 3 (3) | 0.22 | 0.03 | | 0.16 |  | 0.16 | 0.10 | 0.33 |
| CpG 91 | 115 | 2 (2) | 2 (2) | 0.03 | 0.78 | | 0.89 |  | 0.17 | 0.07 | 0.27 |
| CpG 92 | 104 | 17 (12) | 13 (10) | -0.13 | 0.23 | | 0.61 |  | -0.01 | 0.89 | 0.92 |
| CpG 93.94 | 112 | 8 (4) | 8 (5) | 0.11 | 0.28 | | 0.65 |  | 0.04 | 0.67 | 0.85 |
| CpG 95.96 | 113 | 3 (1) | 4 (2) | 0.20 | 0.05 | | 0.23 |  | 0.28 | 0.01 | 0.13 |
| CpG 97 | 112 | 5 (2) | 4 (2) | -0.10 | 0.33 | | 0.69 |  | -0.18 | 0.08 | 0.27 |
| CpG 98.99.112.113 | 113 | 4 (2) | 4 (3) | -0.07 | 0.51 | | 0.86 |  | -0.08 | 0.40 | 0.80 |
| CpG 100 | 113 | 3 (2) | 4 (3) | 0.07 | 0.46 | | 0.81 |  | -0.13 | 0.20 | 0.53 |
| CpG 101.102 | 111 | 5 (2) | 5 (2) | -0.14 | 0.19 | | 0.54 |  | -0.12 | 0.24 | 0.60 |
| CpG 103 | 105 | 7 (9) | 9 (12) | 0.05 | 0.65 | | 0.89 |  | 0.02 | 0.84 | 0.90 |
| CpG 104 | 113 | 2 (1) | 3 (2) | 0.19 | 0.06 | | 0.27 |  | 0.27 | 0.01 | 0.13 |
| CpG 105.106 | 113 | 8 (2) | 9 (3) | 0.28 | 0.00 | | 0.06 |  | 0.19 | 0.06 | 0.24 |
| CpG 107.108 | 113 | 7 (5) | 11 (9) | 0.27 | 0.01 | | 0.06 |  | 0.18 | 0.07 | 0.27 |
| CpG 109.110.111 | 113 | 5 (2) | 5 (1) | -0.02 | 0.84 | | 0.89 |  | 0.01 | 0.91 | 0.94 |
| CpG 114.121.138 | 109 | 10 (5) | 11 (5) | F(1.94)=0.502 | 0.48 | | 0.83 |  | r=0.09 | 0.37 | 0.80 |
| CpG 122.123 | 113 | 4 (5) | 5 (6) | 0.09 | 0.37 | | 0.70 |  | 0.06 | 0.52 | 0.80 |
| CpG 124.125.126 | 113 | 2 (1) | 3 (1) | 0.10 | 0.32 | | 0.69 |  | 0.20 | 0.04 | 0.24 |
| CpG 128.129.130 | 107 | 5 (2) | 5 (2) | 0.11 | 0.28 | | 0.65 |  | 0.12 | 0.22 | 0.56 |
| CpG 131 | 95 | 7 (4) | 6 (3) | -0.10 | 0.39 | | 0.72 |  | -0.06 | 0.60 | 0.84 |
| CpG 132 | 105 | 4 (1) | 4 (2) | 0.18 | 0.07 | | 0.28 |  | 0.23 | 0.03 | 0.22 |
| CpG 133 | 107 | 3 (3) | 2 (4) | -0.02 | 0.83 | | 0.89 |  | 0.11 | 0.27 | 0.63 |
| CpG 141.142 | 113 | 3 (2) | 3 (3) | -0.04 | 0.67 | | 0.89 |  | -0.13 | 0.19 | 0.52 |
| CpG 143 | 113 | 4 (1) | 4 (2) | -0.06 | 0.56 | | 0.88 |  | -0.05 | 0.60 | 0.84 |
| CpG 144 | 113 | 8 (3) | 10 (5) | 0.24 | 0.01 | | 0.06 |  | 0.24 | 0.03 | 0.21 |
| CpG 145.146 | 113 | 3 (2) | 3 (2) | 0.02 | 0.85 | | 0.89 |  | 0.00 | 0.98 | 0.98 |
| CpG 147 | 90 | 7 (3) | 7 (2) | -0.04 | 0.74 | | 0.89 |  | 0.04 | 0.74 | 0.87 |
| CpG 149 | 113 | 11 (4) | 12 (3) | 0.18 | 0.07 | | 0.27 |  | 0.08 | 0.41 | 0.80 |
| CpG 150 | 113 | 7 (4) | 7 (2) | 0.04 | 0.66 | | 0.89 |  | 0.08 | 0.45 | 0.80 |
| CpG 151.160.168 | 113 | 10 (5) | 9 (4) | -0.11 | 0.28 | | 0.65 |  | -0.03 | 0.78 | 0.88 |
| CpG 153.154.155.156.157 | 113 | 8 (3) | 8 (2) | -0.06 | 0.57 | | 0.88 |  | 0.00 | 0.97 | 0.98 |
| CpG 158.159 | 113 | 9 (5) | 7 (4) | -0.11 | 0.29 | | 0.65 |  | 0.02 | 0.88 | 0.92 |
| CpG 161.162.163 | 113 | 8 (3) | 9 (2) | 0.18 | 0.06 | | 0.27 |  | 0.04 | 0.68 | 0.85 |
| CpG 164.165 | 113 | 9 (3) | 10 (3) | 0.22 | 0.03 | | 0.16 |  | 0.07 | 0.46 | 0.80 |
| CpG 166.167 | 113 | 22 (6) | 21 (5) | -0.04 | 0.66 | | 0.89 |  | 0.02 | 0.83 | 0.90 |
| **CpG 169** | 113 | 11 (3) | 13 (3) | 0.29 | < 0.01 | | **0.04** |  | 0.07 | 0.45 | 0.80 |
| ^a^ Different N for each site is due to missing data | | | | | | | | | | | |
| ^b^ Standarized *β* from permutation tests of Student *t-*tests for group comparisons. For CpG sites with normally distributed DNA methylation data, ANCOVAs were used (F-values) | | | | | | | | | | | |
| ^C^ Standarized *β* from linear regression models were used for the analyses of CM load associations used. For CpG sites with normally distributed DNA methylation data, results from multiple linear regressions are shown. | | | | | | | | | | | |
| ^d^ The false discovery rate was used to correct for multiple comparison | | | | | | | | | | | |
| CpG sites that show significant DNA methylation associations with CM are highlighted in bold | | | | | |  |  |  |  |  |  |
